# Supplementary material for: BIGKnock: fine-mapping gene-based associations via knockoff analysis of biobank-scale data
Source: Genome Biol. 2023 Feb 13;24:24. doi: 10.1186/s13059-023-02864-6 (PMC9926792; doi:10.1186/s13059-023-02864-6)
Supplement: Supplementary file 1 — Additional file 1: Figures S1-22 and Table S1-5. [file 13059_2023_2864_MOESM1_ESM.pdf]

Supplemental Material. BIGKnock: Fine-mapping gene-based associations via knockoff analysis of biobank-scale data

Shiyang Ma[1,2], Chen Wang[1], Atlas Khan[3], Linxi Liu[4], James Dalgleish[1], Krzysztof Kiryluk[3], Zihuai He[5,6], Iuliana Ionita-Laza[1,#]

[1] Department of Biostatistics, Columbia University, New York, NY
[2] Clinical Research Institute, Shanghai Jiao Tong University School of Medicine, Shanghai, China
[3] Division of Nephrology, Department of Medicine, Vagelos College of Physicians & Surgeons, Columbia University, New York, NY
[4] Department of Statistics, University of Pittsburgh, Pittsburgh, PA
[5] Quantitative Sciences Unit, Department of Medicine, Stanford University, Stanford, CA
[6] Department of Neurology and Neurological Sciences, Stanford University, Stanford, CA
[#]Correspondance: ii2135@columbia.edu

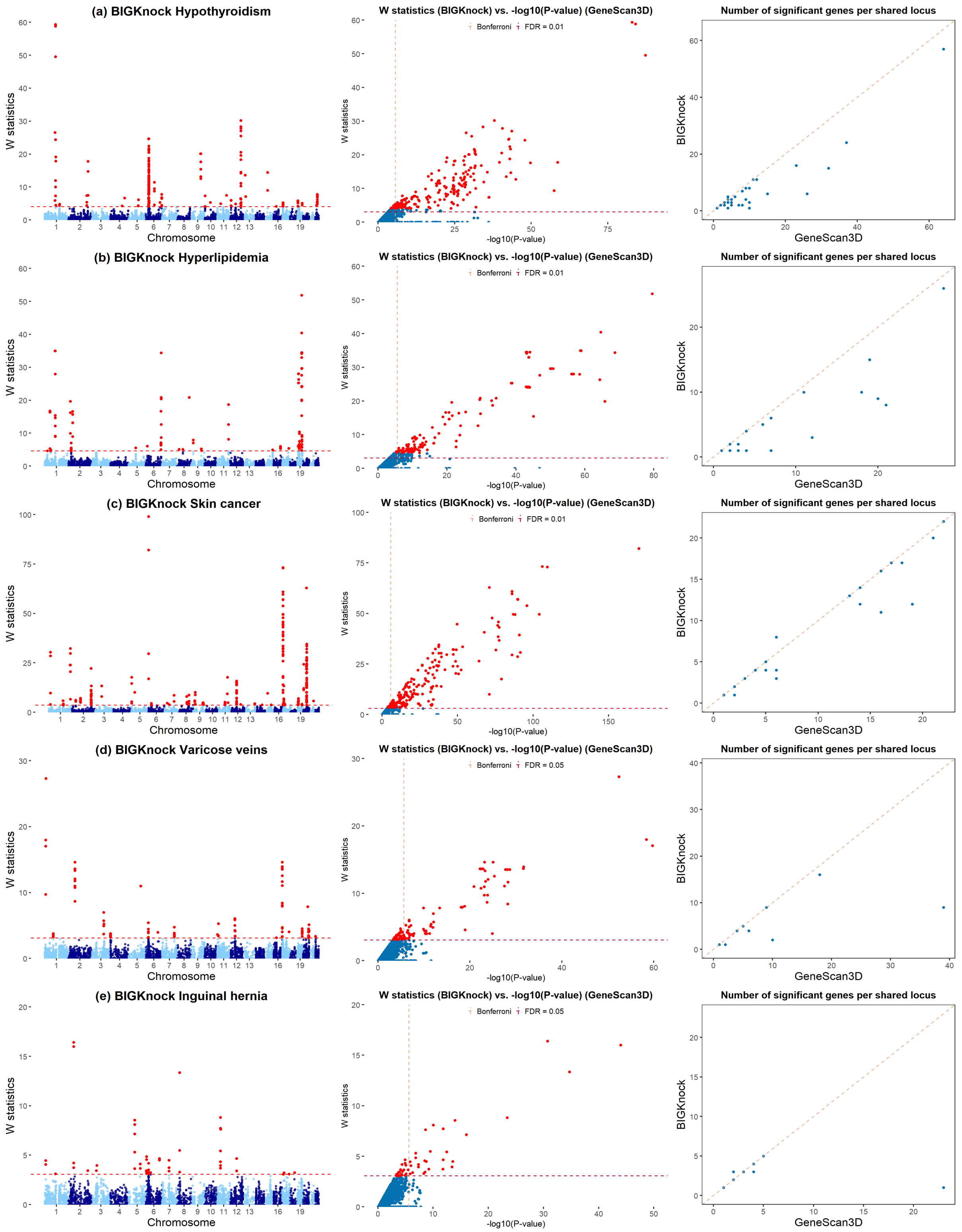

Figure S1: **Applications to UK Biobank binary traits (2)**. (a)-(e), Manhattan plots for BIGKnock, Scatter plot of W knockoff statistics (BIGKnock) vs. $-\log_{10}$(p value) (GeneScan3D), and Scatter plot of the number of significant genes per locus between conventional GeneScan3D and BIGKnock are shown for (a) Hypothyroidism, (b) Hyperlipidemia, (c) Skin cancer, (d) Varicose veins and (e) Inguinal hernia. The dashed lines in the left and middle panels show the significance thresholds defined by Bonferroni correction (for p-values) and by false discovery rate (FDR; for W statistic).

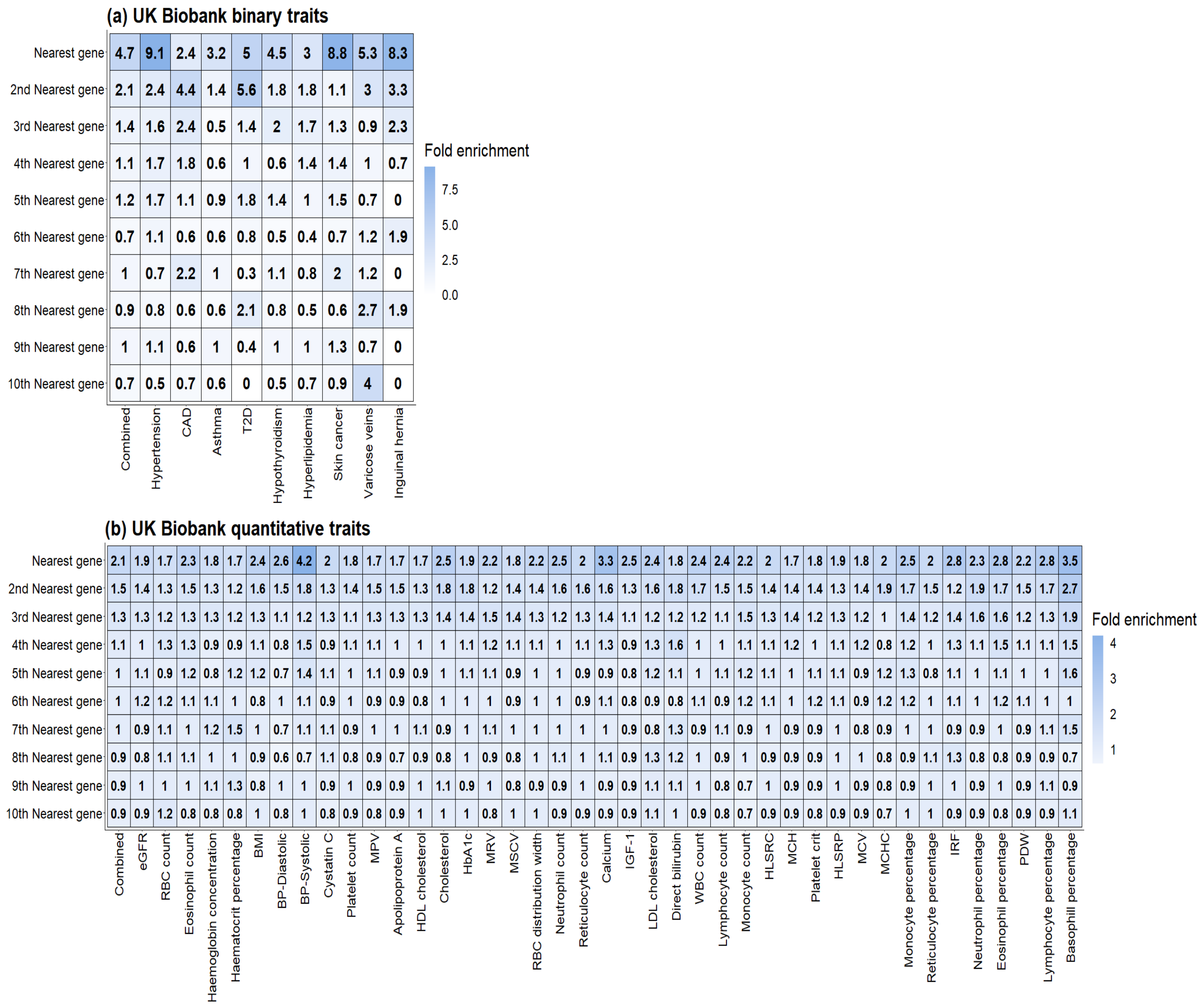

Figure S2: **Enrichment of BIGKnock significant genes among genes closest to the lead GWAS variant at shared loci between BIGKnock and GeneScan3D**. Enrichment of BIGKnock significant genes for (a) the nine combined binary traits and each binary trait separately; and (b) the 41 combined quantitative traits and each quantitative trait separately.

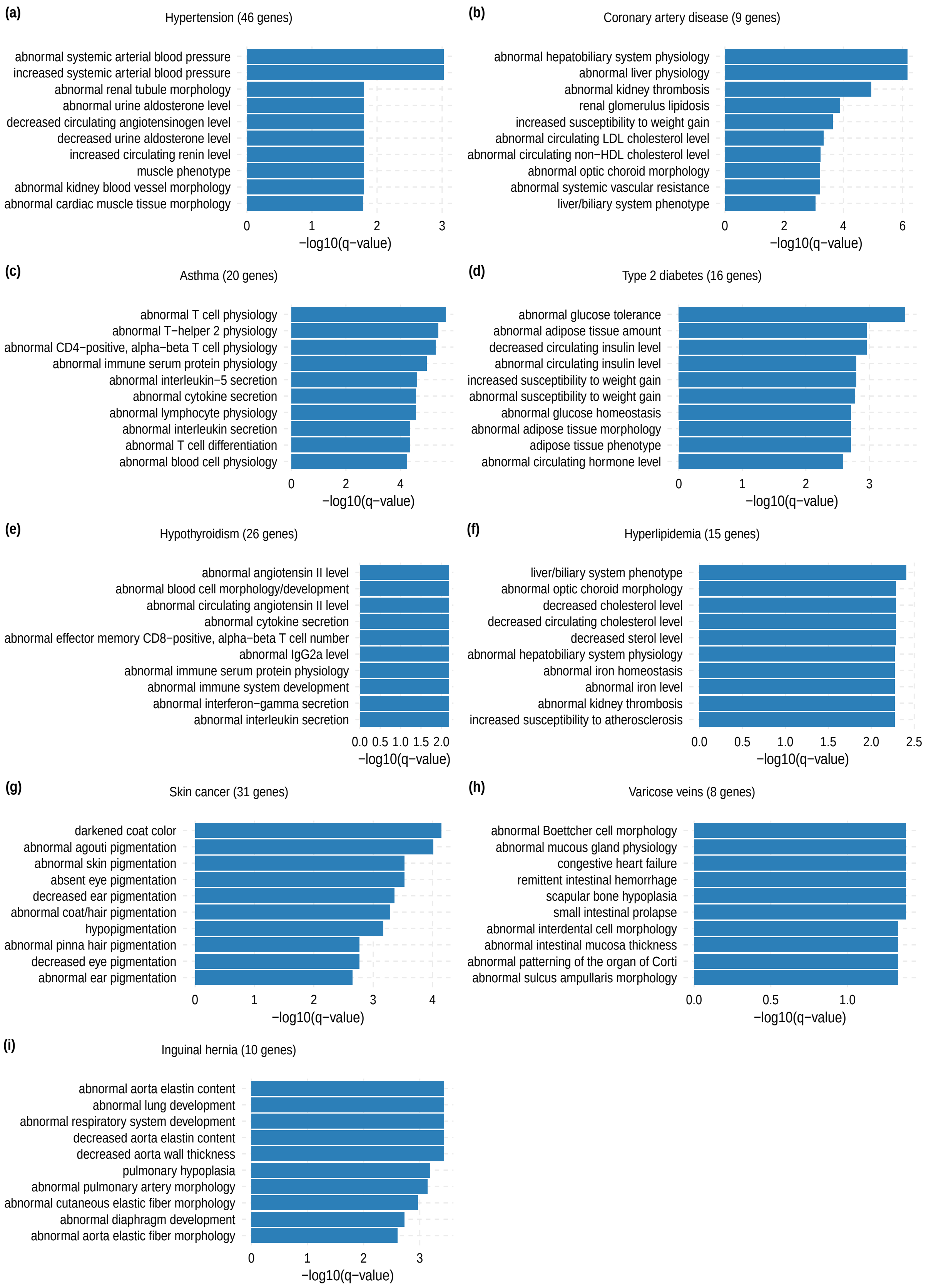

Figure S3: **Mouse phenotype enrichment analyses for nine binary traits in ToppFun.** The top 10 mouse phenotypes in terms of q-value are shown for each trait. The number of effector BIGKnock genes used in these analyses is indicated for each trait.

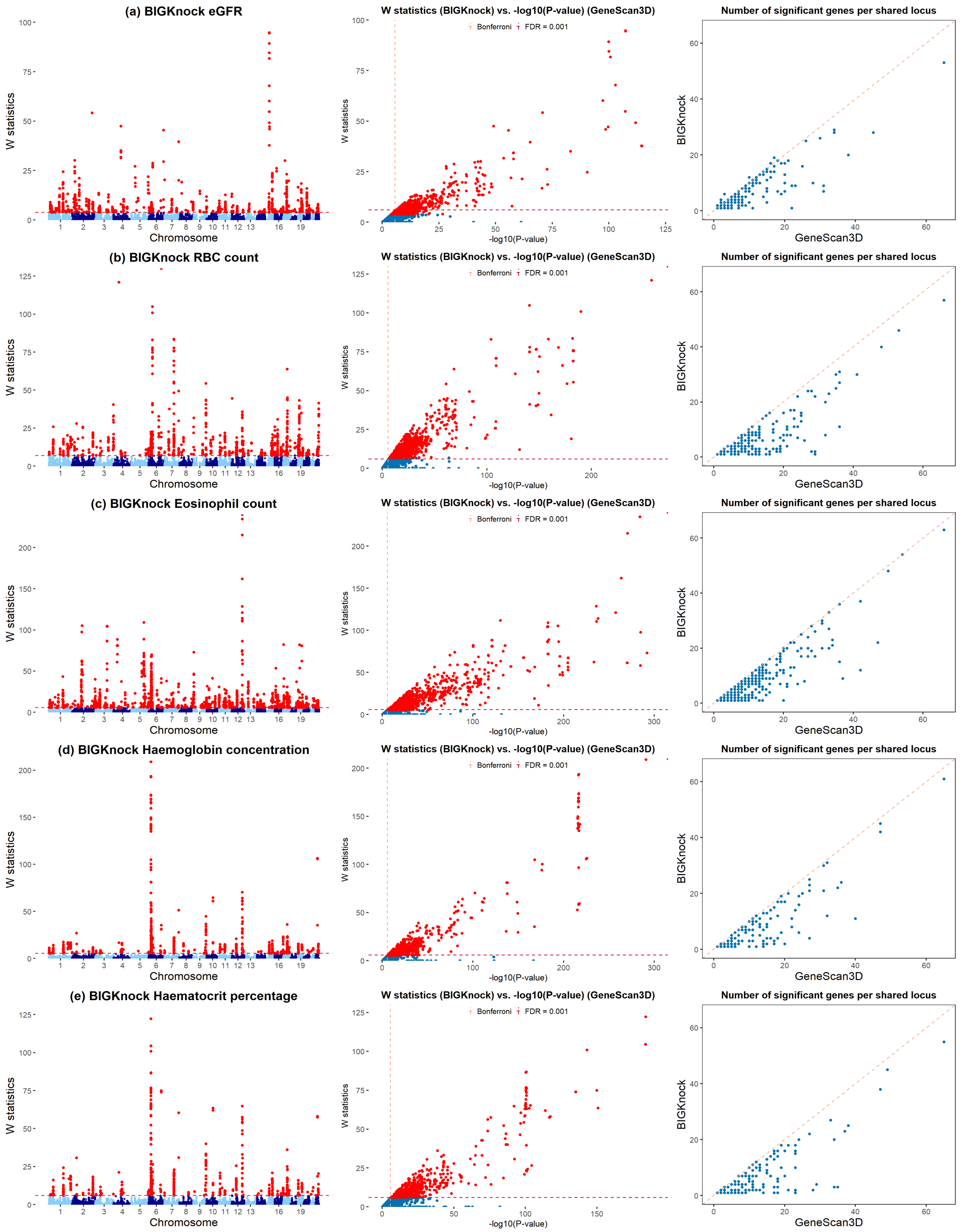

Figure S4: **Applications to UK Biobank quantitative traits (1)**. a-e, Manhattan plots for BIGKnock, Scatter plot of W knockoff statistics (BIGKnock) vs. $-\log_{10}$(p value) (GeneScan3D), and Scatter plot of the number of significant genes per locus between conventional GeneScan3D and BIGKnock are shown for (a) eGFR, (b) RBC count, (c) Eosinophil count, (d) Haemoglobin concentration, and (e) Haematocrit percentage. The dashed lines in the left and middle panels show the significance thresholds defined by Bonferroni correction (for p-values) and by false discovery rate (FDR; for W statistic).

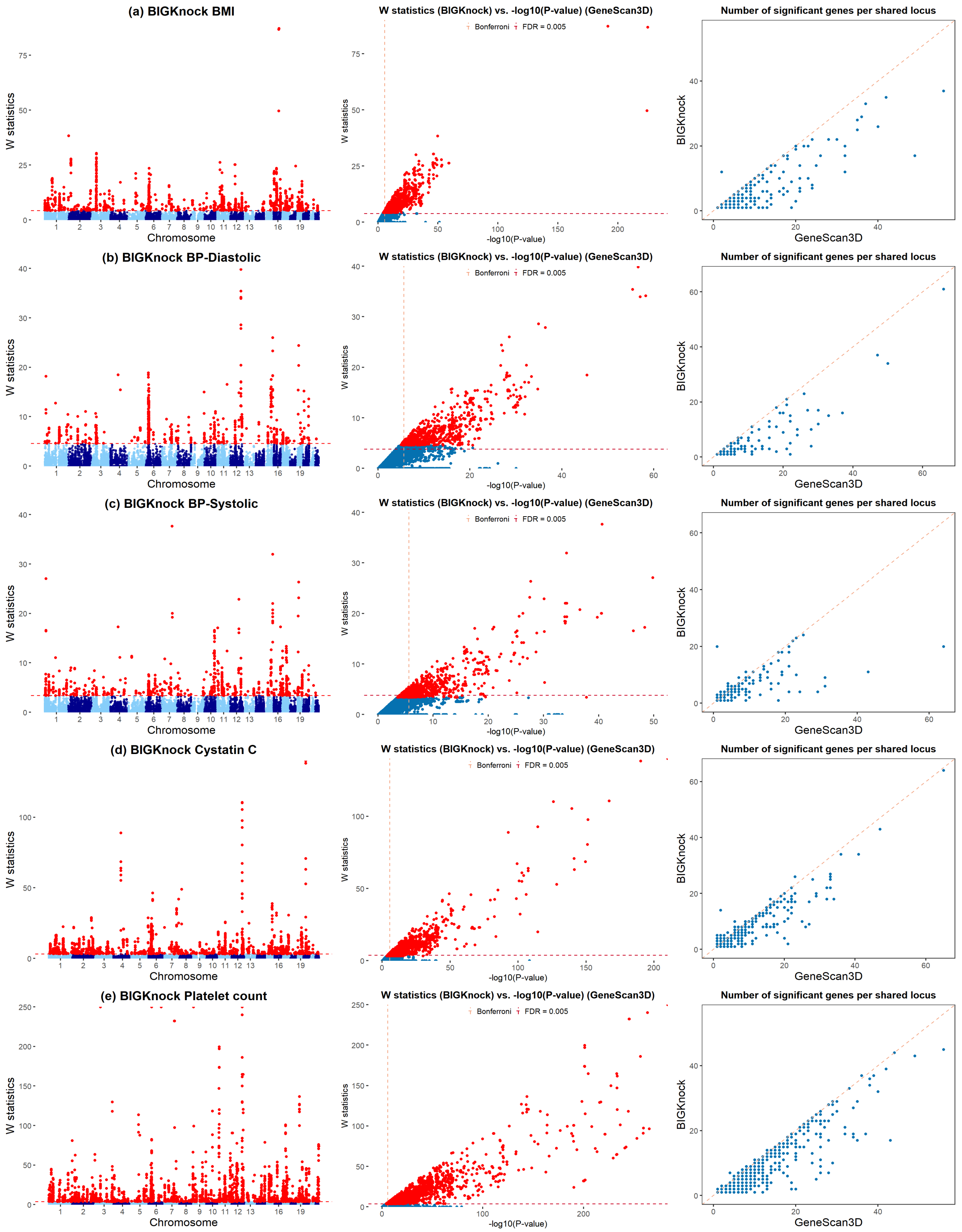

Figure S5: **Applications to UK Biobank quantitative traits (2)**. a-e, Manhattan plots for BIGKnock, Scatter plot of W knockoff statistics (BIGKnock) vs. $-\log_{10}$(p value) (GeneScan3D), and Scatter plot of the number of significant genes per locus between conventional GeneScan3D and BIGKnock are shown for (a) BMI, (b) BP-Diastolic, (c) BP-Systolic, (d) Cystatin C, and (e) Platelet count. The dashed lines in the left and middle panels show the significance thresholds defined by Bonferroni correction (for p-values) and by false discovery rate (FDR; for W statistic).

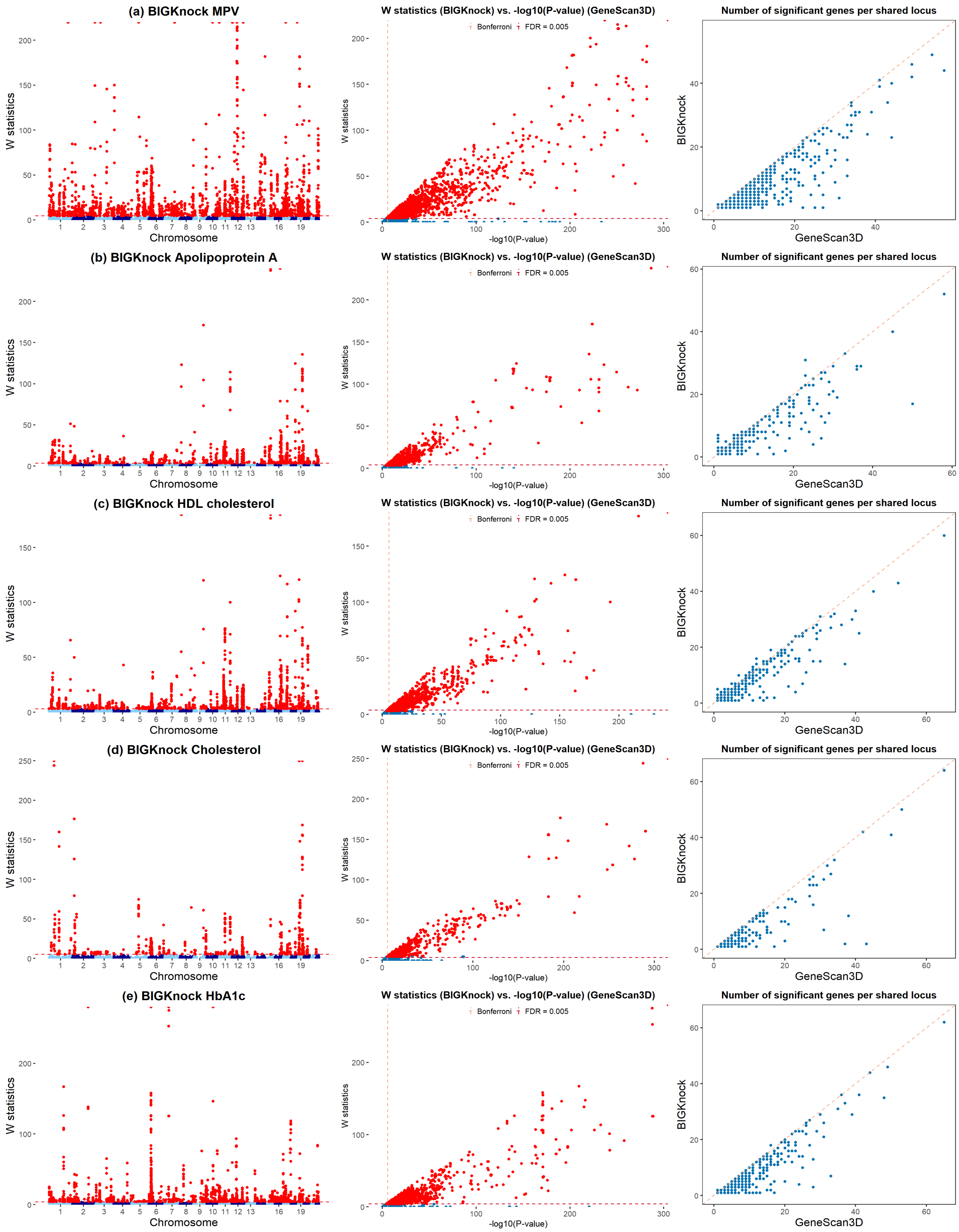

Figure S6: **Applications to UK Biobank quantitative traits (3)**. a-e, Manhattan plots for BIGKnock, Scatter plot of W knockoff statistics (BIGKnock) vs. $-\log_{10}$(p value) (GeneScan3D), and Scatter plot of the number of significant genes per locus between conventional GeneScan3D and BIGKnock are shown for (a) MPV, (b) Apolipoprotein A, (c) HDL cholesterol, (d) Cholesterol, and (e) HbA1c. The dashed lines in the left and middle panels show the significance thresholds defined by Bonferroni correction (for p-values) and by false discovery rate (FDR; for W statistic).

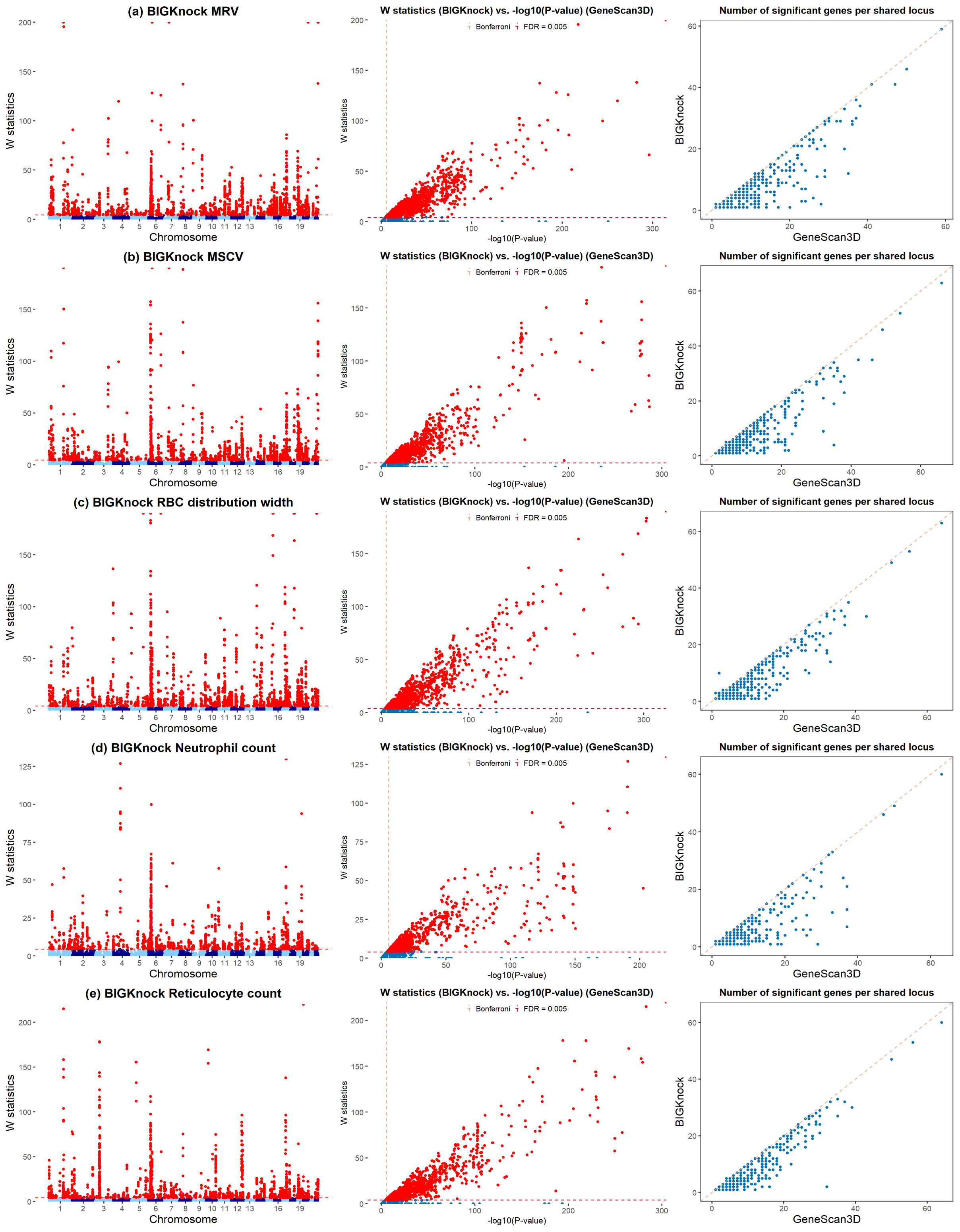

Figure S7: **Applications to UK Biobank quantitative traits (4)**. a-e, Manhattan plots for BIGKnock, Scatter plot of W knockoff statistics (BIGKnock) vs. $-\log_{10}$(p value) (GeneScan3D), and Scatter plot of the number of significant genes per locus between conventional GeneScan3D and BIGKnock are shown for (a) MRV, (b) MSCV, (c) RBC distribution width, (d) Neutrophil count, and (e) Reticulocyte count. The dashed lines in the left and middle panels show the significance thresholds defined by Bonferroni correction (for p-values) and by false discovery rate (FDR; for W statistic).

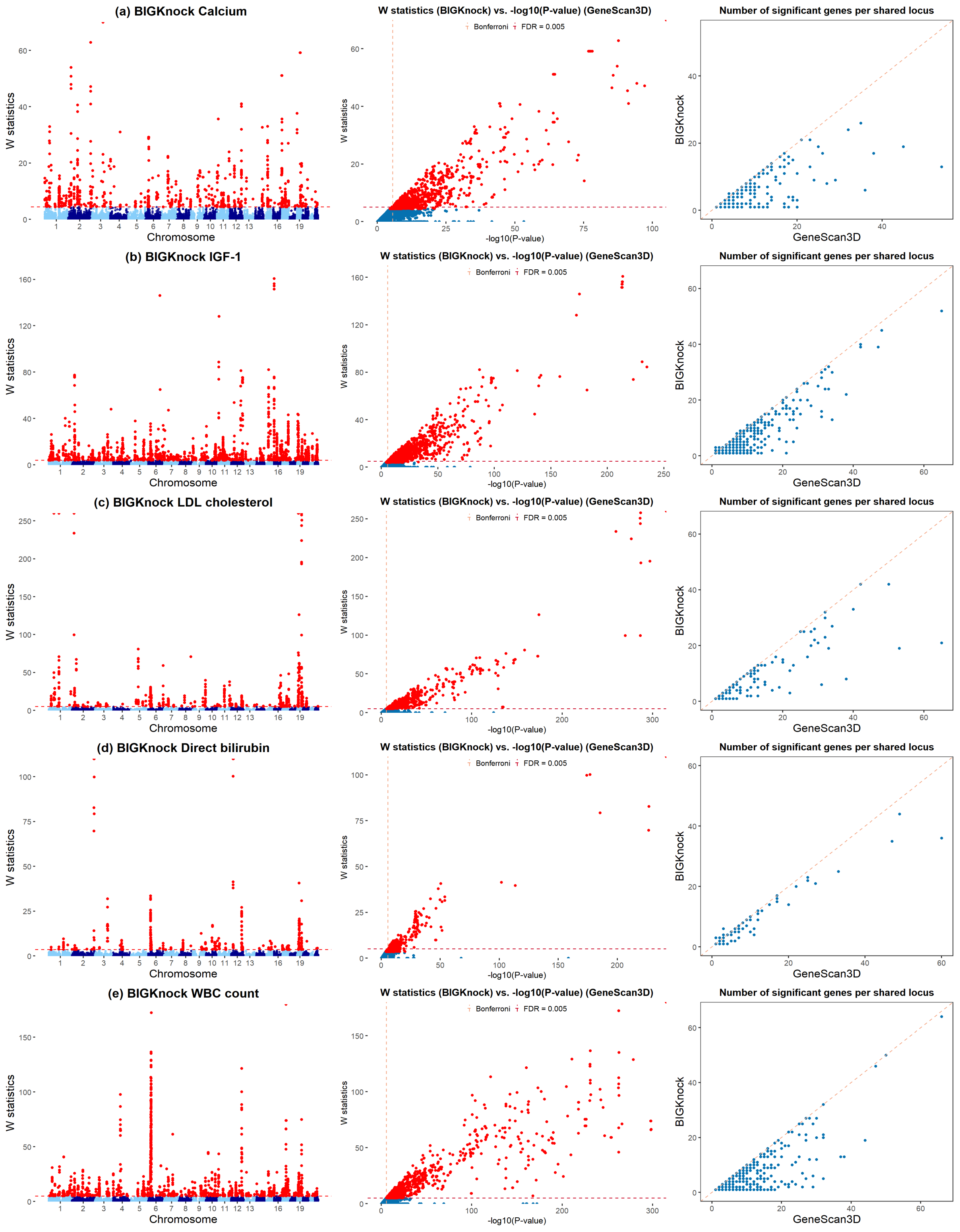

Figure S8: **Applications to UK Biobank quantitative traits (5)**. a-e, Manhattan plots for BIGKnock, Scatter plot of W knockoff statistics (BIGKnock) vs. $-\log_{10}$(p value) (GeneScan3D), and Scatter plot of the number of significant genes per locus between conventional GeneScan3D and BIGKnock are shown for (a) Calcium, (b) IGF-1, (c) LDL cholesterol, (d) Direct bilirubin, and (e) WBC count. The dashed lines in the left and middle panels show the significance thresholds defined by Bonferroni correction (for p-values) and by false discovery rate (FDR; for W statistic).

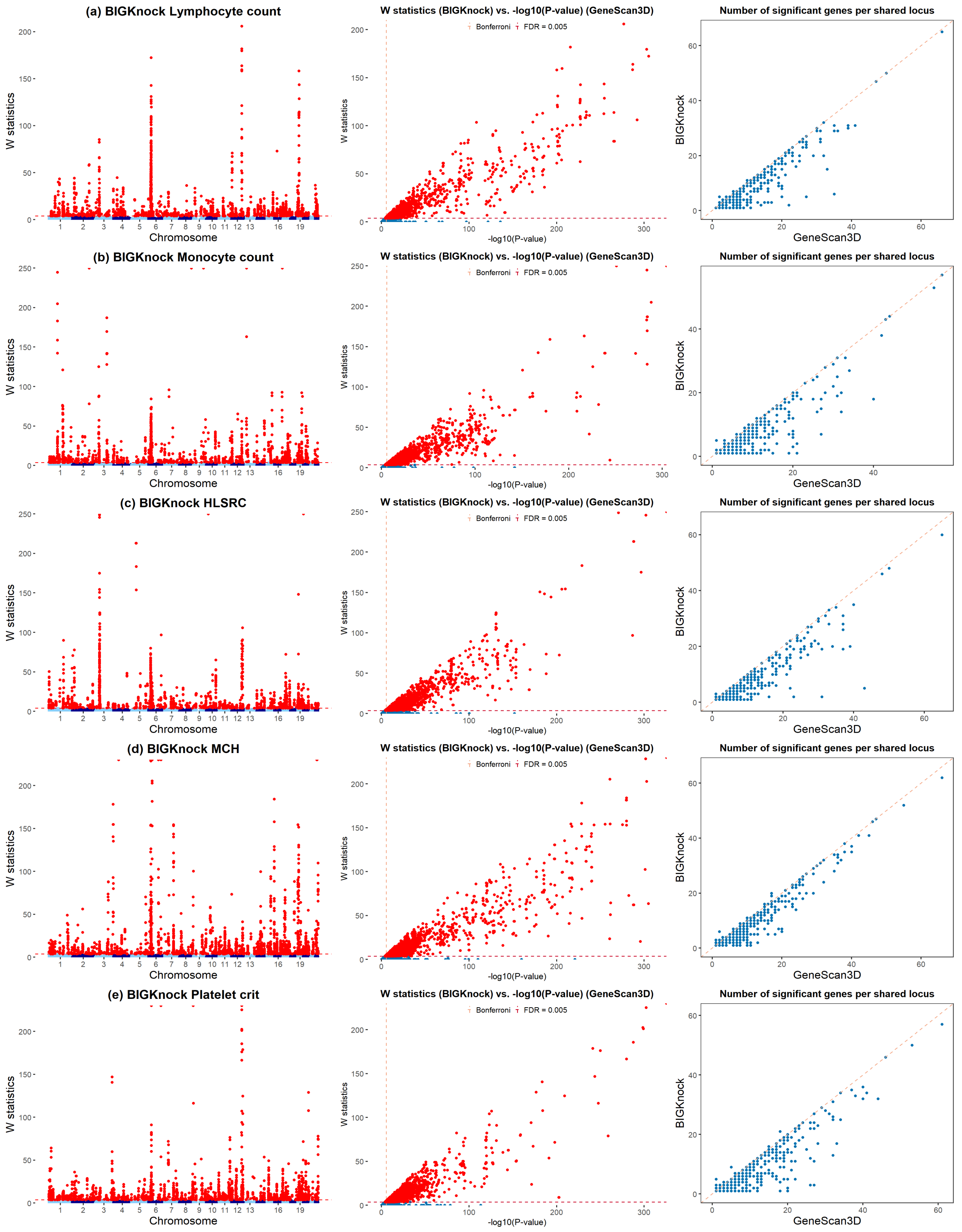

Figure S9: **Applications to UK Biobank quantitative traits (6)**. a-e, Manhattan plots for BIGKnock, Scatter plot of W knockoff statistics (BIGKnock) vs. $-\log_{10}$(p value) (GeneScan3D), and Scatter plot of the number of significant genes per locus between conventional GeneScan3D and BIGKnock are shown for (a) Lymphocyte count, (b) Monocyte count, (c) HLSRC, (d) MCH, and (e) Platelet crit. The dashed lines in the left and middle panels show the significance thresholds defined by Bonferroni correction (for p-values) and by false discovery rate (FDR; for W statistic).

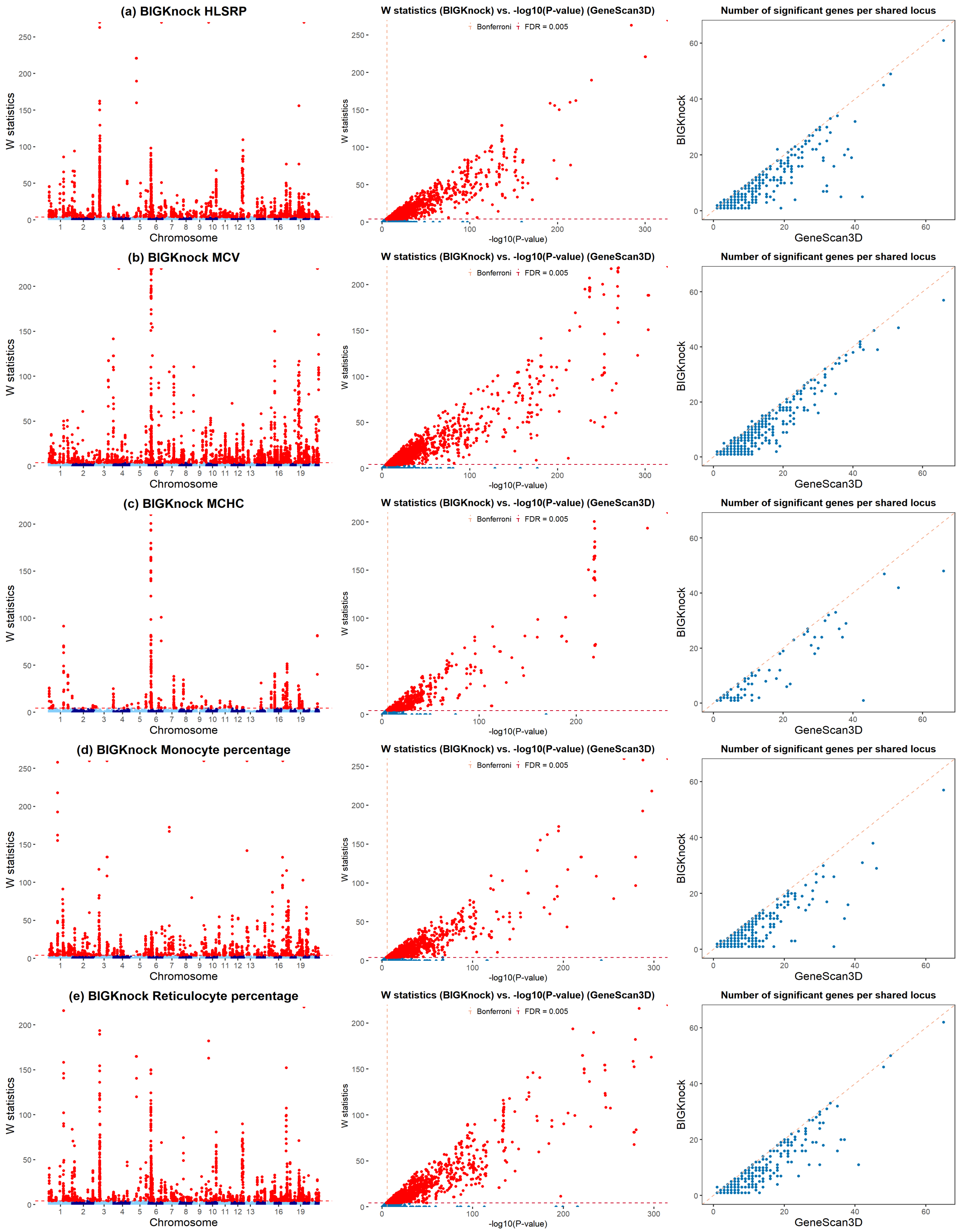

Figure S10: **Applications to UK Biobank quantitative traits (7).** a-e, Manhattan plots for BIGKnock, Scatter plot of W knockoff statistics (BIGKnock) vs. $-\log_{10}$(p value) (GeneScan3D), and Scatter plot of the number of significant genes per locus between conventional GeneScan3D and BIGKnock are shown for (a) HLSRP, (b) MCV, (c) MCHC, (d) Monocyte percentage, and (e) Reticulocyte percentage. The dashed lines in the left and middle panels show the significance thresholds defined by Bonferroni correction (for p-values) and by false discovery rate (FDR; for W statistic).

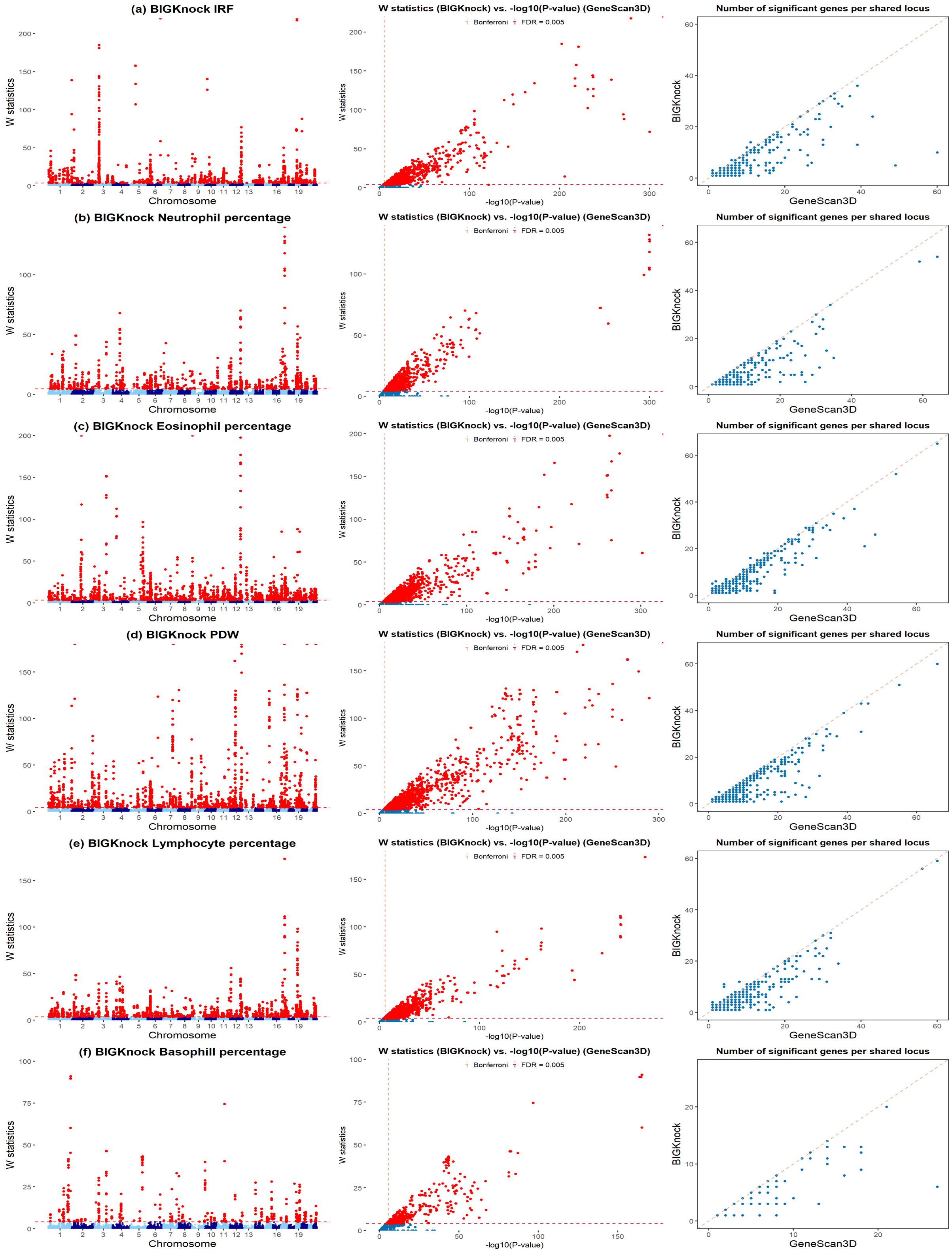

Figure S11: **Applications to UK Biobank quantitative traits (8)**. a-c, Manhattan plots for BIGKnock, Scatter plot of W knockoff statistics (BIGKnock) vs. $-\log_{10}$(p value) (GeneScan3D), and Scatter plot of the number of significant genes per locus between conventional GeneScan3D and BIGKnock are shown for (a) IRF, (b) Neutrophil percentage, (c) Eosinophil percentage, (d) PDW, (e) Lymphocyte percentage, and (f) Basophill percentage. The dashed lines in the left and middle panels show the significance thresholds defined by Bonferroni correction (for p-values) and by false discovery rate (FDR; for W statistic).

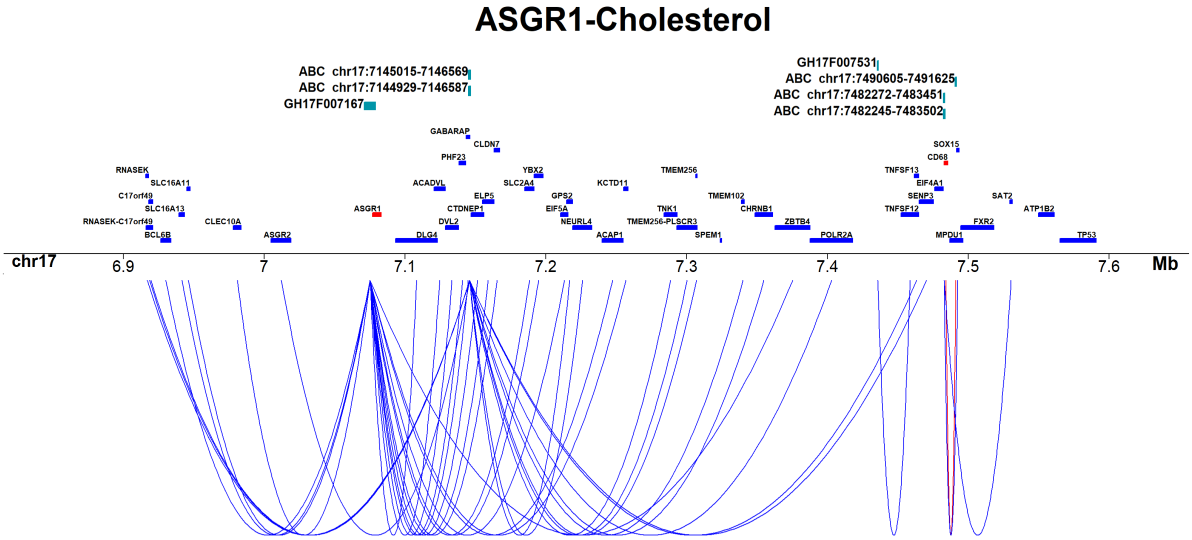

Figure S12: **Visualization of gene-enhancer interactions of significant genes at the ASGR1-Cholesterol locus.** Gene-enhancer interactions for 43 GeneScan3D significant genes at the ASGR1-Cholesterol locus, with the two BIGKnock significant genes (*ASGR1* and *CD68*) shown in red. The interaction between BIGKnock significant gene *CD68* and ABC enhancer chr17:7,490,605-7,491,625 is shown in red; other 35 gene-enhancer links are shown in blue (See Supplementary Table 26).

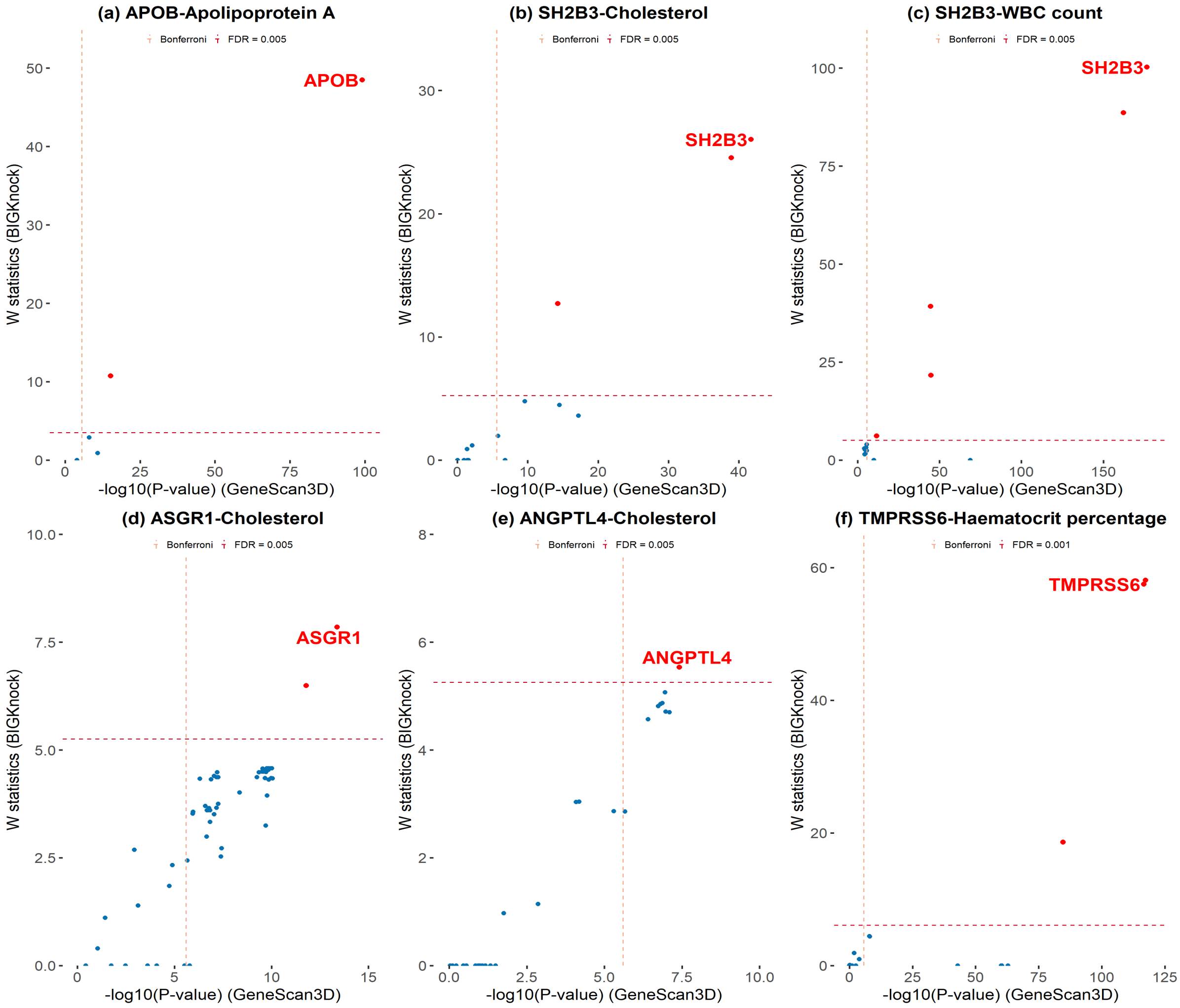

Figure S13: **Selected loci that pinpoint effector genes identified by Backman et al. [52]** Scatter plots of W knockoff statistics (BIGKnock) vs. $-\log_{10}$(p value) (GeneScan3D) for 6 selected loci that pinpoint effector genes identified by Backman et al. [52]. The effector genes are labeled in red. The dashed lines show the significance thresholds defined by Bonferroni correction (for p-values) and by false discovery rate (FDR; for W statistic).

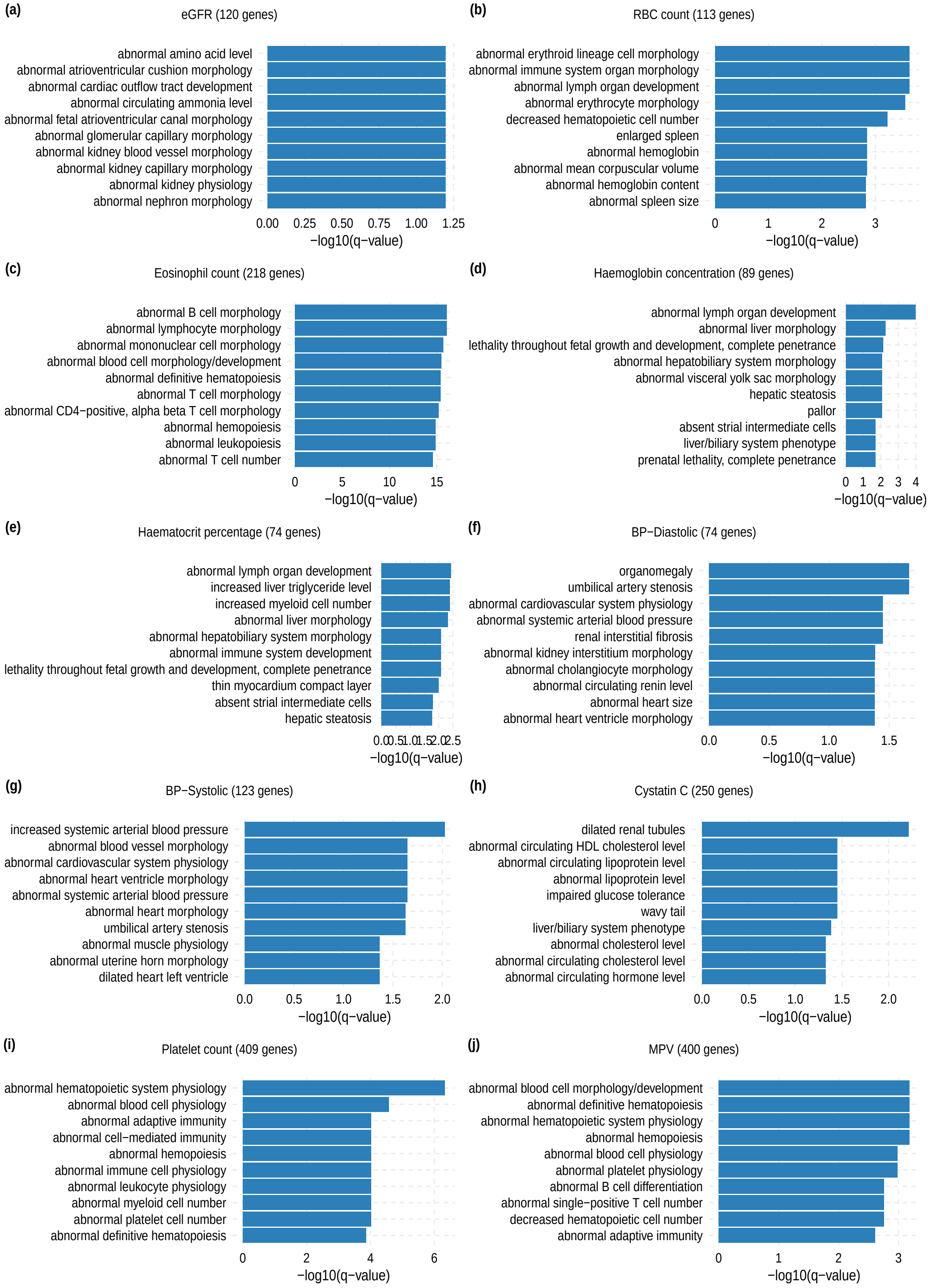

Figure S14: **Mouse phenotype enrichment analyses for 10 quantitative traits in ToppFun.** The top 10 mouse phenotypes in terms of q-value are shown for each trait. The number of effector BIGKnock genes used in these analyses are indicated for each trait.

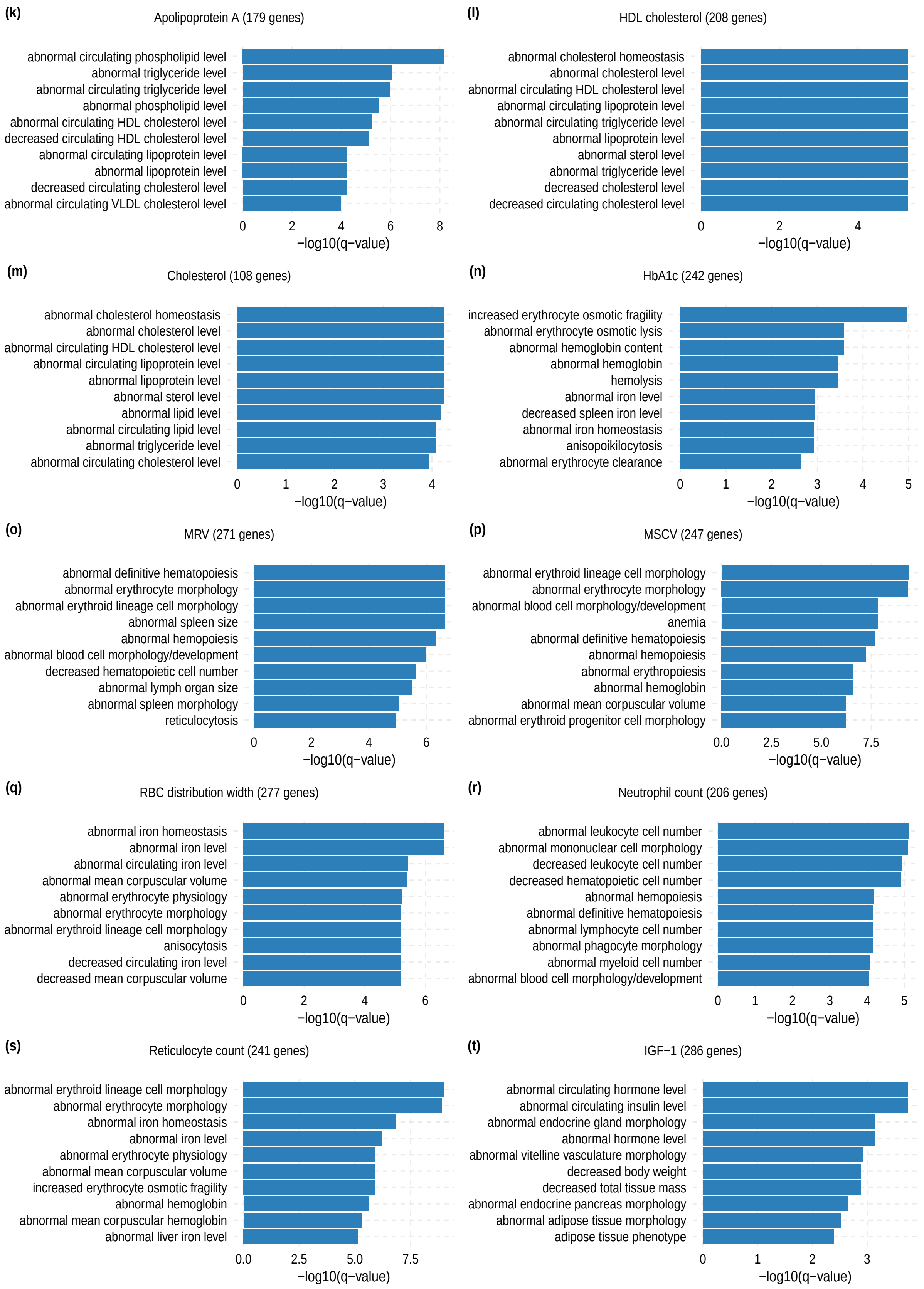

Figure S15: **Mouse phenotype enrichment analyses for 10 quantitative traits in ToppFun.** The top 10 mouse phenotypes in terms of q-value are shown for each trait. The number of effector BIGKnock genes used in these analyses are indicated for each trait.

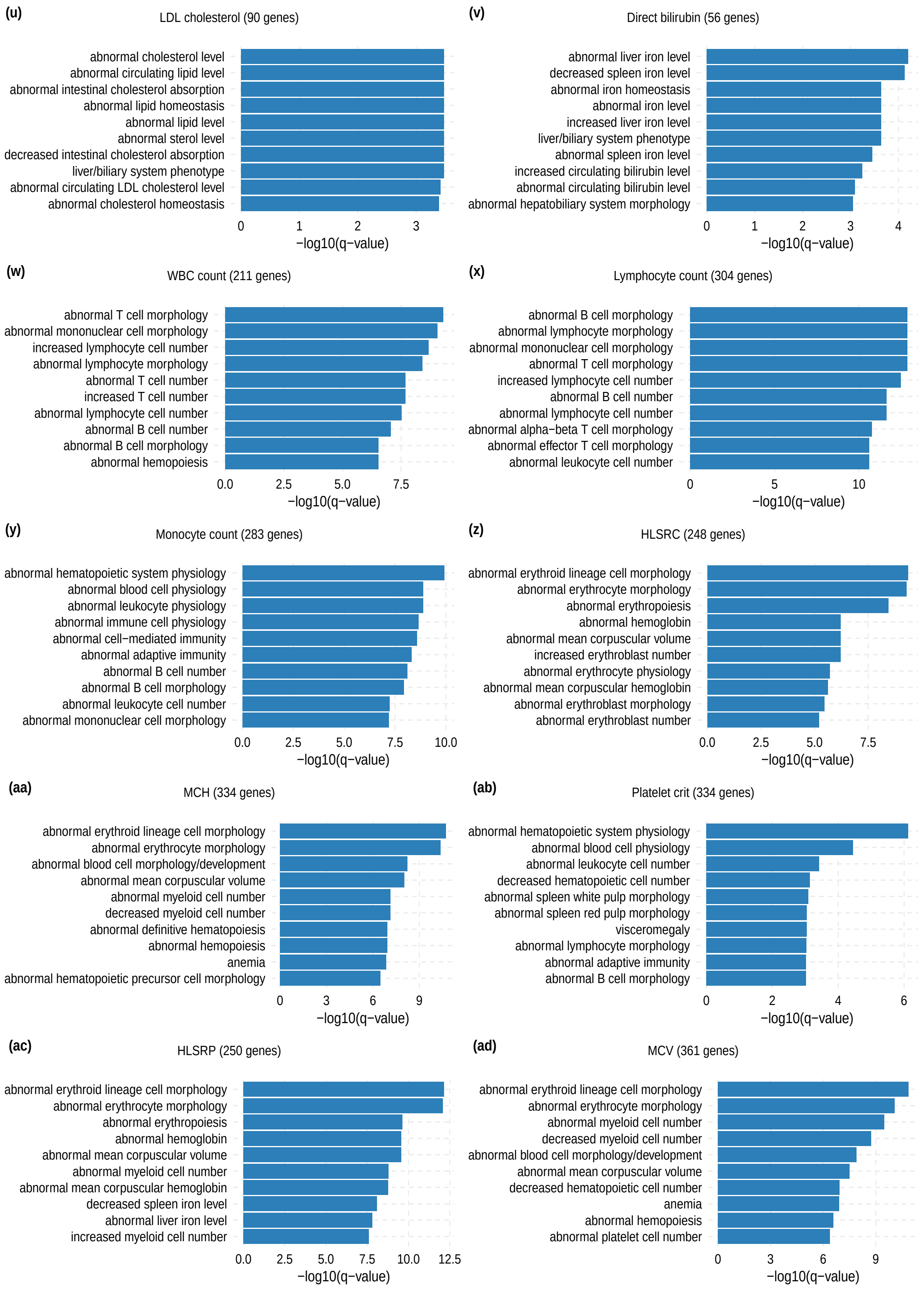

Figure S16: **Mouse phenotype enrichment analyses for 10 quantitative traits in ToppFun.** The top 10 mouse phenotypes in terms of q-value are shown for each trait. The number of effector BIGKnock genes used in these analyses are indicated for each trait.

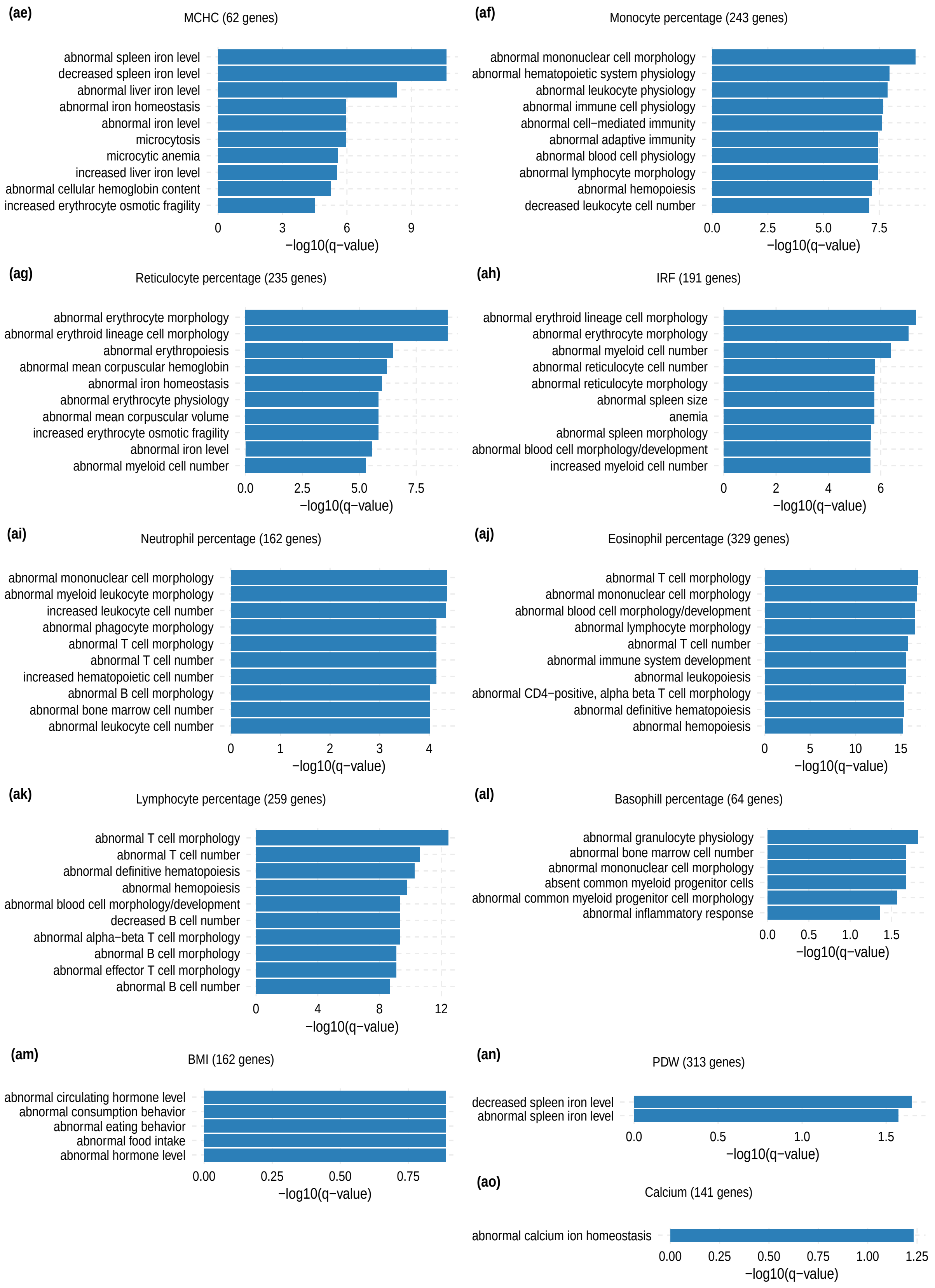

Figure S17: **Mouse phenotype enrichment analyses for 11 quantitative traits in ToppFun.** The top 10 mouse phenotypes in terms of q-value are shown for each trait. The number of effector BIGKnock genes used in these analyses are indicated for each trait.

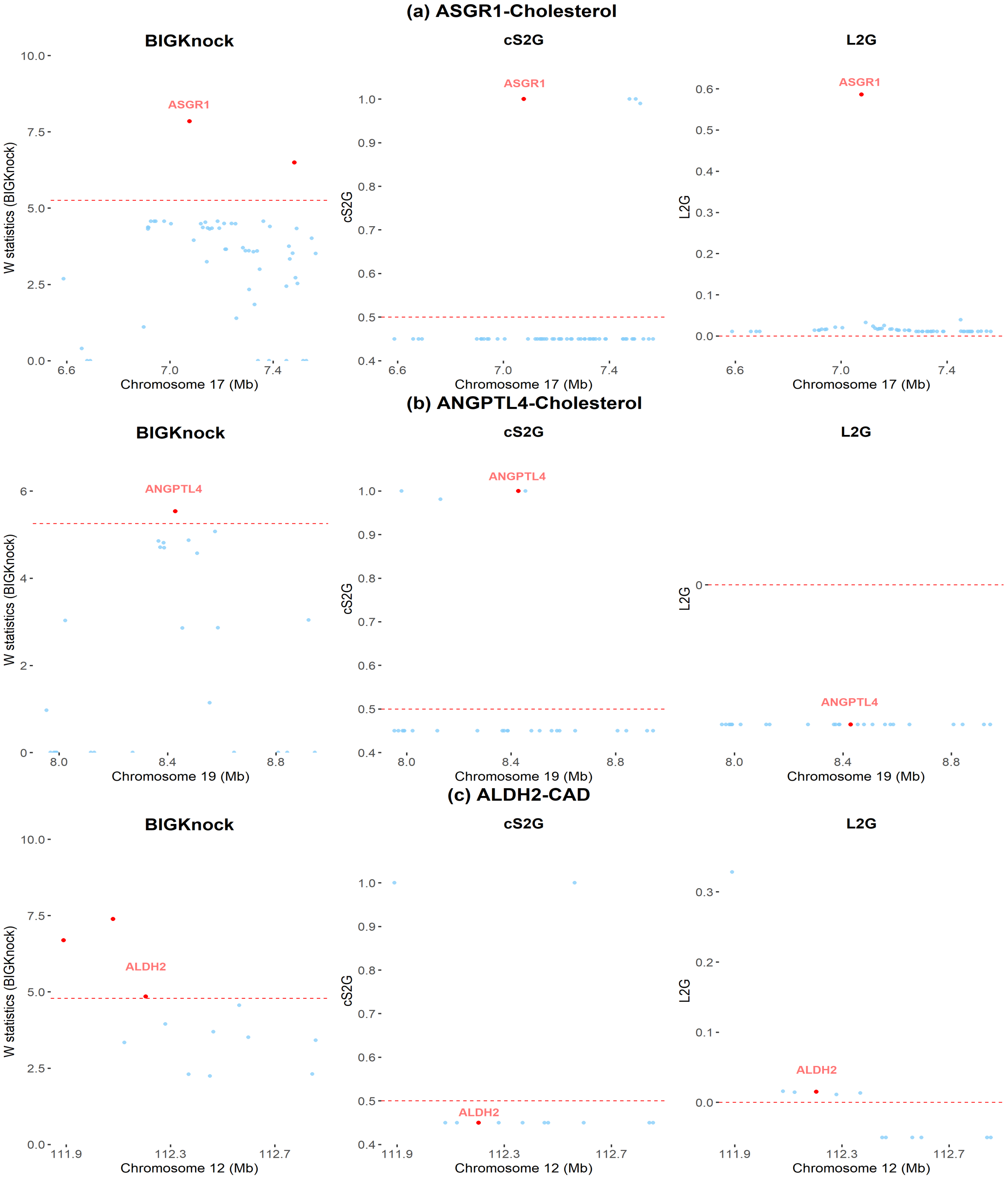

Figure S18: **BIGKnock, cS2G and L2G results at three loci containing known causal genes (ASGR1-Cholesterol, ANGPTL4-Cholesterol and ALDH2-CAD).** W knockoff statistics, cS2G scores, and L2G scores are shown for genes at the 1Mb loci containing known causal genes. The putative causal gene at each locus is labeled.

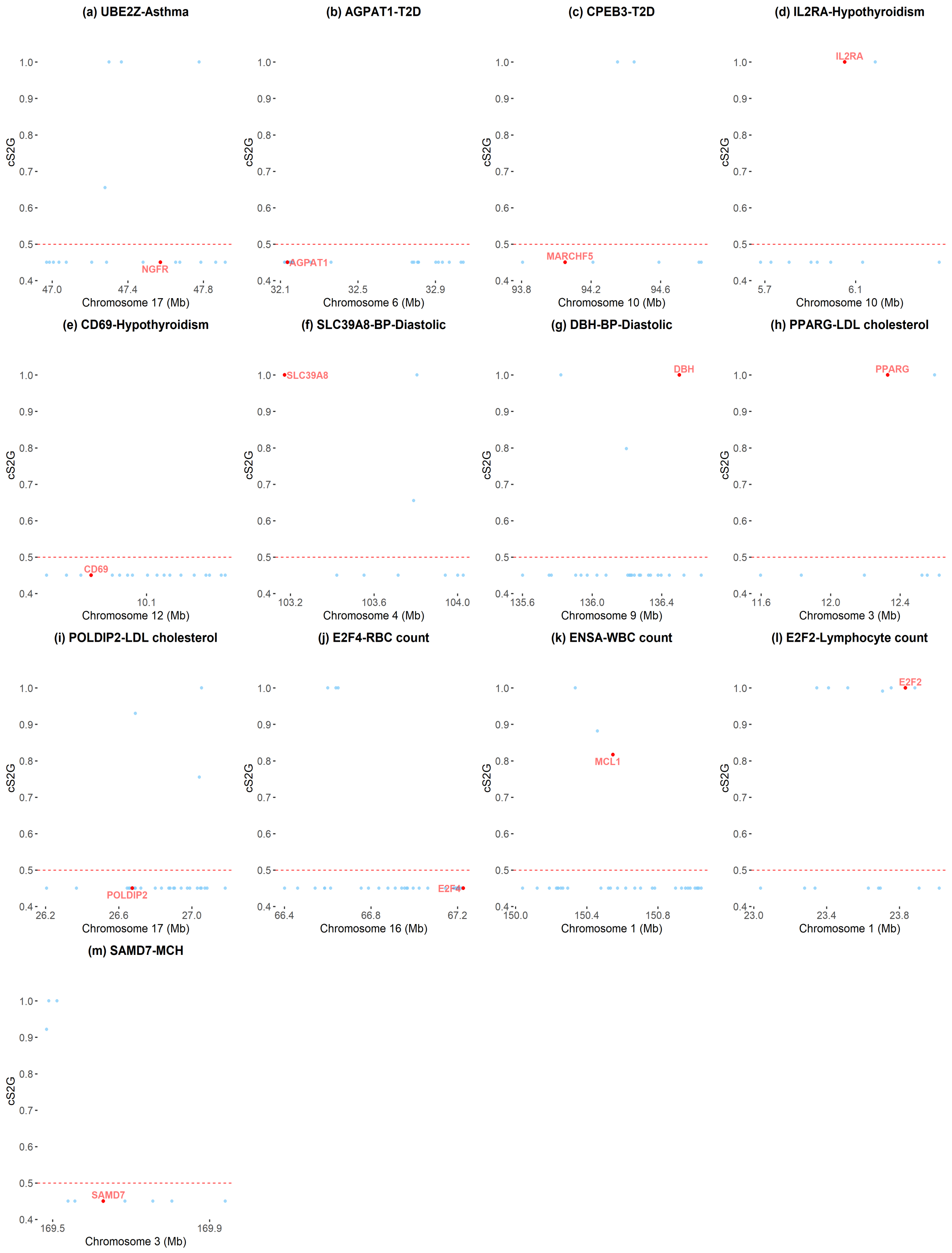

Figure S19: **cS2G scores of putative causal genes at selected loci for UK Biobank binary traits and quantitative traits.** cS2G scores for genes at selected loci for (a) Asthma, (b-c) T2D, (d-e) Hypothyroidism, (f-g) BP-Diastolic, (h-i) LDL cholesterol, (j) RBC count, (k) WBC count, (l) Lymphocyte count and (m) MCH. Loci are named according to the most significant gene in BIGKnock. The dashed line corresponds to the recommended threshold (0.5) for cS2G. Genes which do not have a cS2G score are shown just below the 0.5 threshold. The labeled gene corresponds to the putative causal gene at the locus as discussed in the Results section.

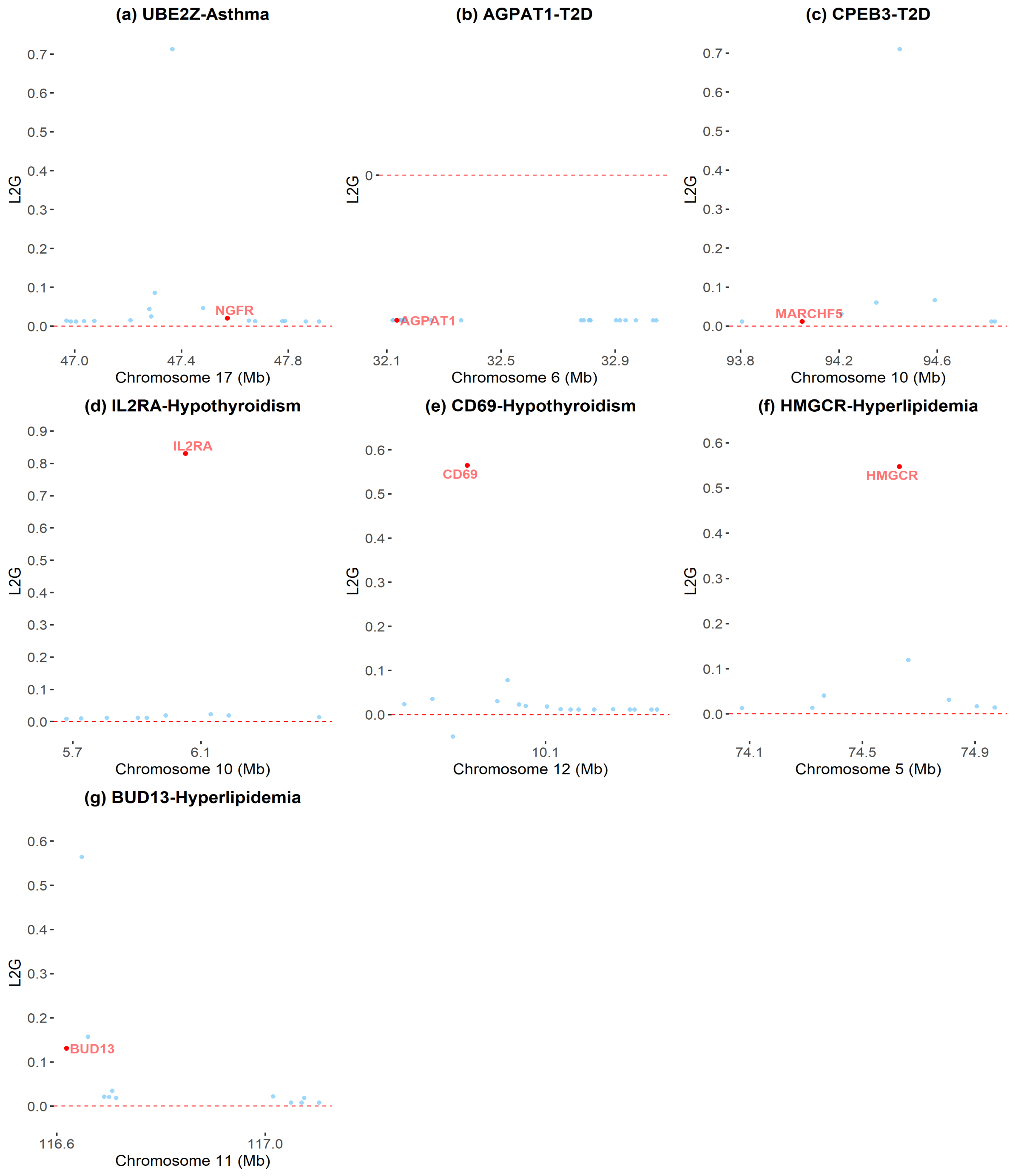

Figure S20: **L2G scores of putative causal genes at selected loci for UK Biobank binary traits**. L2G scores for genes at selected loci for (a) Asthma, (b-c) Type 2 diabetes (T2D), (d-e) Hypothyroidism and (f-g) Hyperlipidemia. Loci are named according to the most significant gene in BIGKnock. The dashed line corresponds to the recommended threshold (0) for L2G. Genes which do not have a L2G score are shown just below the 0 threshold. The labeled gene corresponds to the putative causal gene at the locus as discussed in the Results section.

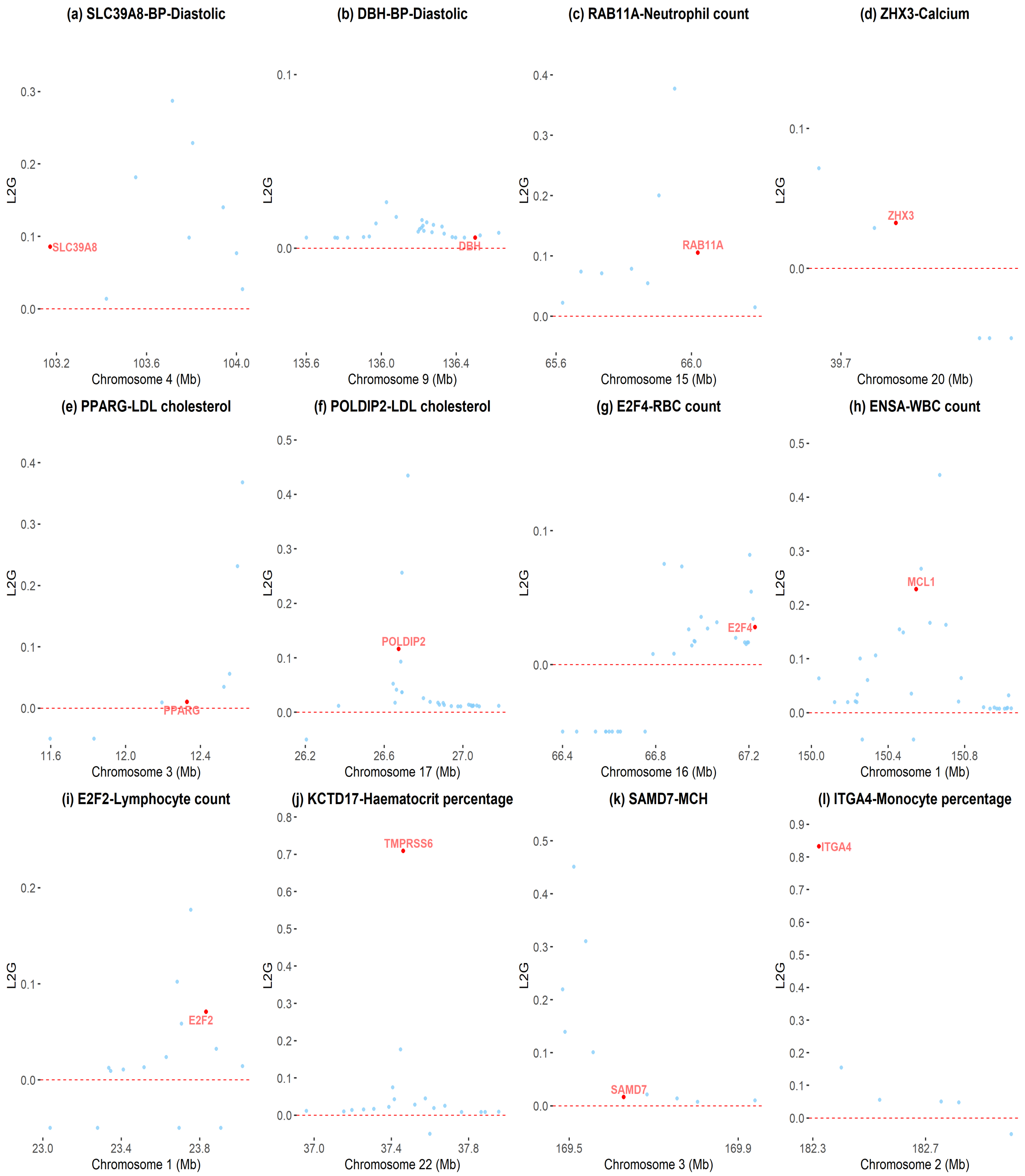

Figure S21: **L2G scores of putative causal genes at selected loci for UK Biobank quantitative traits**. L2G scores for genes at selected loci for selected loci of (a-b) BP-Diastolic, (c) Neutrophil count, (d) Calcium, (e-f) LDL cholesterol, (g) RBC count, (h) WBC count, (i) Lymphocyte count, (j) Haematocrit percentage, (k) MCH and (l) Monocyte percentage. Loci are named according to the most significant gene in BIGKnock. The dashed line corresponds to the recommended threshold (0) for L2G. Genes which do not have a L2G score are shown just below the 0 threshold. The labeled gene corresponds to the putative causal gene at the locus as discussed in the Results section.

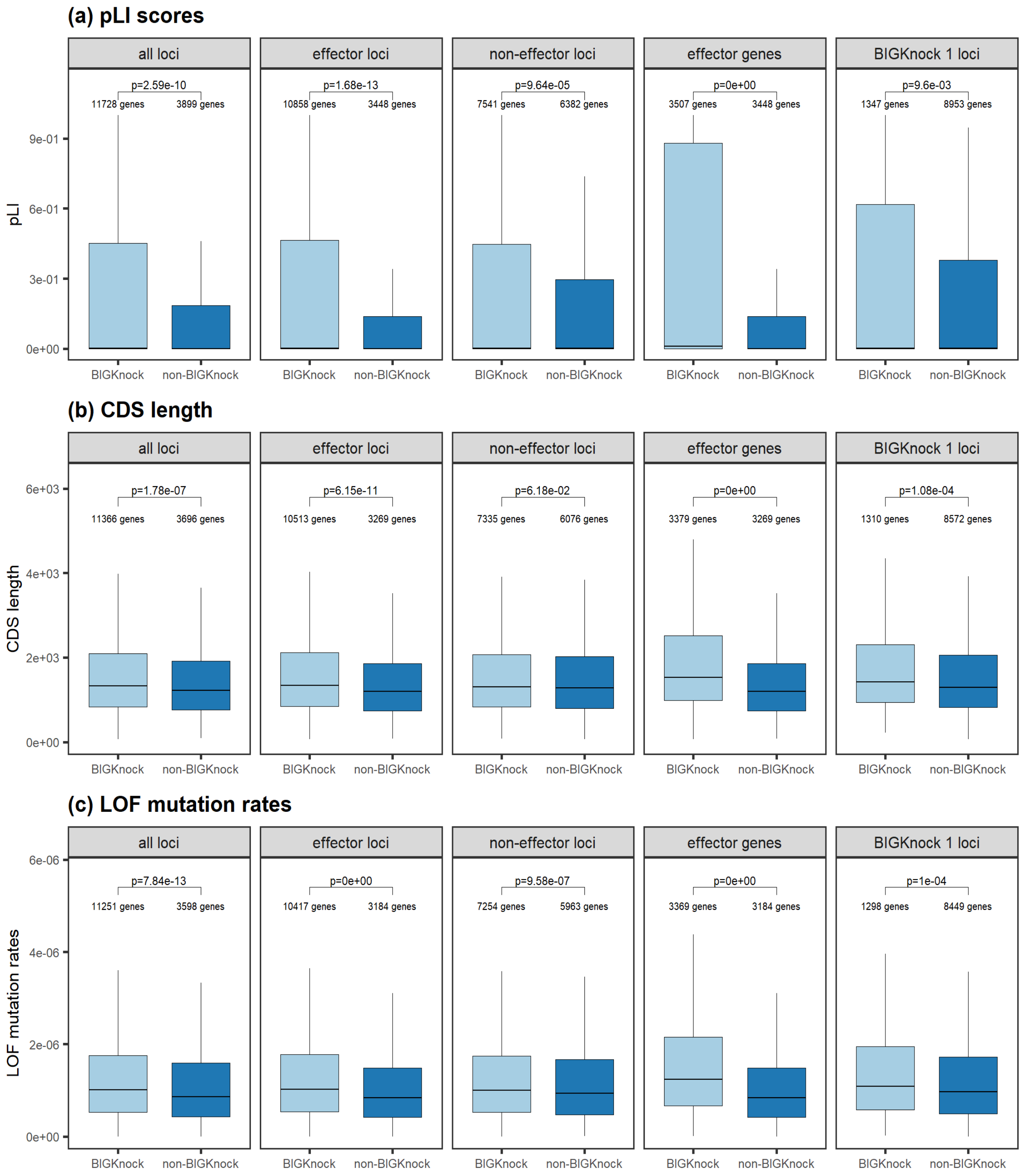

Figure S22: **Characteristics of prioritized genes.** (a) pLI scores, (b) CDS length and (c) LOF mutation rates for BIGKnock significant genes and genes that are never selected by BIGKnock across a variety of binary and quantitative traits considered here at all loci, effector BIGKnock loci (loci that have effector genes), non-effector loci, effector BIGKnock genes vs. genes that are never significant under the BIGKnock test across all traits, and BIGKnock 1 loci (loci where BIGKnock prioritizes only one gene). The number of genes in each group are shown, along with p-values from a Kolmogorov-Smirnov test.

Table S1: **Summary of nine selected UK Biobank binary traits.**

**Binary traits**	Phecode	*n*	# cases/# controls (case:control ratio)
Hypertension	401	404,265	90,714/313,551 (1:3.5)
Coronary artery disease (CAD)	411	404,186	35,400/368,786 (1:10.4)
Asthma	495	395,745	29,934/365,811 (1:12.2)
Type 2 diabetes (T2D)	250.2	403,490	21,964/381,526 (1:17.4)
Hypothyroidism	244	402,019	17,616/384,403 (1:21.8)
Hyperlipidemia	272.1	404,854	42,350/362,504 (1:8.6)
Skin cancer	172	404,805	28,254/376,551 (1:13.3)
Varicose veins	454	375,266	12,986/362,280 (1:27.9)
Inguinal hernia	550.1	368,255	17,976/350,279 (1:19.5)

Table S2: **The number of significant loci/genes associated with each binary trait for GeneScan3D and BIGKnock, and the number of loci shared between them.**

**Binary traits**	**GeneScan3D**	**BIGKnock**	**Shared loci**	**FDR threshold**
Hypertension	81 loci/ 491 genes	64 loci/ 333 genes	61 loci	0.01
Coronary artery disease	26 loci/ 188 genes	15 loci/ 108 genes	15 loci	0.01
Asthma	27 loci/ 335 genes	25 loci/ 238 genes	25 loci	0.01
Type 2 diabetes	36 loci/ 164 genes	36 loci/ 120 genes	31 loci	0.05
Hypothyroidism	44 loci/ 356 genes	35 loci/ 205 genes	35 loci	0.01
Hyperlipidemia	31 loci/ 243 genes	24 loci/ 143 genes	24 loci	0.01
Skin cancer	34 loci/ 322 genes	32 loci/ 252 genes	31 loci	0.01
Varicose veins	14 loci/ 136 genes	11 loci/ 107 genes	11 loci	0.05
Inguinal hernia	18 loci/ 60 genes	15 loci/ 49 genes	12 loci	0.05

Table S3: **Summary of 41 selected UK quantitative traits.**

**Quantitative traits**	*n*	Phenocode
estimated glomerular filtration rate (eGFR)	386,253	-
Red blood cell (erythrocyte) count (RBC count)	393,208	30010
Eosinophil count	392,512	30150
Haemoglobin concentration	393208	30020
Haematocrit percentage	393,208	30030
Body Mass Index (BMI)	403,979	21001
Diastolic Blood Pressure Automated Reading (BP-Diastolic)	378,531	4080
Systolic Blood Pressure Automated Reading (BP-Systolic)	378,522	4079
Cystatin C	386,398	30720
Platelet count	393,205	30080
Mean platelet volume (MPV)	393,200	30100
Apolipoprotein A	351,755	30630
HDL cholesterol	353,744	30760
Cholesterol	386,452	30690
Glycated haemoglobin (HbA1c)	386,300	30750
Mean reticulocyte volume (MRV)	386,844	30260
Mean sphered cell volume (MSCV)	386,844	30270
Red blood cell (erythrocyte) distribution width (RBC distribution width)	393,189	30070
Neutrophil count	392,512	30140
Reticulocyte count	386,843	30250
Calcium	353,787	30680
IGF-1	384,356	30770
LDL direct (LDL cholesterol)	385,728	30780
Direct bilirubin	328,800	30660
White blood cell (leukocyte) count (WBC count)	393,204	30000
Lymphocyte count	392,512	30120
Monocyte count	392,512	30130
High light scatter reticulocyte count (HLSRC)	386,855	30300
Mean corpuscular haemoglobin (MCH)	393,205	30050
Platelet crit	393,201	30090
High light scatter reticulocyte percentage (HLSRP)	386,856	30290
Mean corpuscular volume (MCV)	393,206	30040
Mean corpuscular haemoglobin concentration (MCHC)	393,202	30060
Monocyte percentage	392,517	30190
Reticulocyte percentage	386,855	30240
Immature reticulocyte fraction (IRF)	386,856	30280
Neutrophil percentage	392,517	30200
Eosinophil percentage	392,517	30210
Platelet distribution width (PDW)	393,200	30110
Lymphocyte percentage	392,517	30180
Basophill percentage	392,517	30220

Table S4: **The number of significant loci/genes associated with each quantitative trait for GeneScan3D and BIGKnock, and the number of loci shared between them.**

Quantitative traits	GeneScan3D	BIGKnock	Shared loci	FDR threshold
eGFR	273 loci/ 1,483 genes	241 loci/ 1,010 genes	225 loci	0.001
RBC count	423 loci/ 2,980 genes	210 loci/ 1,212 genes	210 loci	0.001
Eosinophil count	475 loci/ 3,794 genes	322 loci/ 2,021 genes	322 loci	0.001
Haemoglobin concentration	242 loci/ 1,741 genes	156 loci/ 875 genes	156 loci	0.001
Haematocrit percentage	248 loci/ 1,743 genes	141 loci/ 754 genes	141 loci	0.001
BMI	442 loci/ 2,767 genes	278 loci/ 1,300 genes	272 loci	0.005
BP-Diastolic	221 loci/ 1,498 genes	128 loci/ 677 genes	128 loci	0.005
BP-Systolic	205 loci/ 1,208 genes	204 loci/ 865 genes	171 loci	0.005
Cystatin C	409 loci/ 2,550 genes	434 loci/ 2,092 genes	372 loci	0.005
Platelet count	732 loci/ 5,242 genes	640 loci/ 3,768 genes	635 loci	0.005
MPV	767 loci/ 5,293 genes	644 loci/ 3,496 genes	642 loci	0.005
Apolipoprotein A	304 loci/ 2,362 genes	296 loci/ 1,815 genes	275 loci	0.005
HDL cholesterol	343 loci/ 2,412 genes	349 loci/ 1,974 genes	314 loci	0.005
Cholesterol	220 loci/ 1,812 genes	156 loci/ 998 genes	156 loci	0.005
HbA1c	451 loci/ 3,438 genes	390 loci/ 2,392 genes	380 loci	0.005
MRV	493 loci/ 4,139 genes	402 loci/ 2,661 genes	400 loci	0.005
MSCV	493 loci/ 4,104 genes	380 loci/ 2,479 genes	378 loci	0.005
RBC distribution width	468 loci/ 4,176 genes	409 loci/ 2,896 genes	403 loci	0.005
Neutrophil count	402 loci/ 3,273 genes	312 loci/ 1,865 genes	312 loci	0.005
Reticulocyte count	428 loci/ 3,447 genes	363 loci/ 2,367 genes	358 loci	0.005
Calcium	269 loci/ 1,940 genes	219 loci/ 963 genes	218 loci	0.005
IGF-1	526 loci/ 3,644 genes	427 loci/ 2,324 genes	422 loci	0.005
LDL cholesterol	176 loci/ 1,601 genes	125 loci/ 902 genes	125 loci	0.005
Direct bilirubin	78 loci/ 654 genes	84 loci/ 547 genes	73 loci	0.005
WBC count	485 loci/ 3,893 genes	338 loci/ 1,785 genes	338 loci	0.005
Lymphocyte count	524 loci/ 3,744 genes	432 loci/ 2,539 genes	424 loci	0.005
Monocyte count	478 loci/ 3,514 genes	433 loci/ 2,501 genes	420 loci	0.005
HLSRC	448 loci/ 3,745 genes	371 loci/ 2,412 genes	367 loci	0.005
MCH	547 loci/ 4,493 genes	501 loci/ 3,531 genes	491 loci	0.005
Platelet crit	624 loci/ 4,440 genes	548 loci/ 3,104 genes	535 loci	0.005
HLSRP	457 loci/ 3,743 genes	383 loci/ 2,392 genes	379 loci	0.005
MCV	596 loci/ 4,865 genes	529 loci/ 3,560 genes	519 loci	0.005
MCHC	105 loci/ 1,282 genes	82 loci/ 793 genes	82 loci	0.005
Monocyte percentage	436 loci/ 3,283 genes	357 loci/ 2,099 genes	353 loci	0.005
Reticulocyte percentage	423 loci/ 3,462 genes	355 loci/ 2,250 genes	353 loci	0.005
IRF	304 loci/ 2,760 genes	267 loci/ 1,740 genes	266 loci	0.005
Neutrophil percentage	358 loci/ 2,891 genes	271 loci/ 1,498 genes	269 loci	0.005
Eosinophil percentage	482 loci/ 3,764 genes	447 loci/ 2,944 genes	434 loci	0.005
PDW	541 loci/ 4,064 genes	460 loci/ 2,700 genes	457 loci	0.005
Lymphocyte percentage	416 loci/ 3,154 genes	376 loci/ 2,276 genes	363 loci	0.005
Basophill percentage	115 loci/ 848 genes	88 loci/ 540 genes	86 loci	0.005

Table S5: **Selected loci that pinpoint effector genes identified by Backman et al. [52].**

**BIGKnock locus-trait**	**position (hg19)**	**# GeneScan3D**	**# BIGKnock**	**BIGKnock genes**	**Effector gene**
APOB-Apolipoprotein A	2:20,731,524-21,731,524	4	2	*APOB,TDRD15*	*APOB*
SH2B3-Cholesterol	12:110,868,171-111,868,171	8	3	*FAM109A,PPTC7,SH2B3*	*SH2B3*
SH2B3-WBC count	12:110883036-111883036	9	5	*FAM109A,HVCN1,MYL2 PPTC7,SH2B3*	*SH2B3*
ASGR1-Cholesterol	17:6,569,412-7,569,412	43	2	*ASGR1,CD68*	*ASGR1*
ANGPTL4-Cholesterol	19:7,951,937-8,951,937	9	1	*ANGPTL4*	*ANGPTL4*
TMPRSS6-Haematocrit percentage	22:36962936-37962936	11	3	*KCTD17,MPST,TMPRSS6*	*TMPRSS6*
